# A Case Study of Umbilical Hernia Complicated by the Presence of a Desmoid Tumor

**DOI:** 10.7759/cureus.72960

**Published:** 2024-11-04

**Authors:** Marivel M, Guru Prasad, Samir Ahmad, Santhaseelan R G, Aiswerya Shankar

**Affiliations:** 1 Department of General Surgery, Sree Balaji Medical College and Hospital, Chennai, IND

**Keywords:** aggressive fibromatosis, desmoid fibromatosis, desmoid tumor, tumor excision, umbilical hernia, umbilical herniorrhaphy

## Abstract

Desmoid tumors or aggressive fibromatosis are locally aggressive benign tumors. These arise anywhere in the body but are commonly seen in the anterior abdominal wall. The main treatment choices are continuous surveillance, adjuvant chemotherapy, surgery, and postoperative chemotherapy. However, where needs arise, surgery may be done, specifically wide local excision with adequate clearance. In our case report we show a case of a male patient presenting with umbilical hernia and an incidental discovery of a desmoid tumor that was closely proximate to the herniated sac. The plan of action planned were sac excision followed by tumor excision and then anatomical repair, that is, umbilical herniorrhaphy and postoperative chemotherapy.

## Introduction

Desmoid tumors are rare benign tumors arising from the myoaponeurotic layer. These are said to be mesenchymal in origin [1]. Even though they are benign, they are locally aggressive and mostly nonmetastasizing [2]. These are commonly seen in the anterior abdominal wall, mostly around the umbilicus. The incidence is particularly higher in the infraumbilical region. These are nonencapsulated and firm to hard tumors. WHO has classified this as intermediate locally aggressive tumors [3]. The incidence of desmoid tumors is two to four per million population [1]. The incidence in women is four times higher than in males and is commonly seen between the ages of 15 and 60 years [4,5]. The chances of recurrence in intra-abdominal desmoid tumors are 57%-86% [6]. These are commonly associated with familial adenomatous polyposis (FAP) [1] and Gardner's syndrome [7]. In FAP, the chances of getting a desmoid tumor are higher. These tumors often undergo myxomatous changes but never sarcomatous changes, unlike fibromas. The best course of action in treatment is wide local excision with adequate clearance. There have been many instances where desmoid tumors have been found associated with inguinal hernia [7]. These tumors are associated with a mutation in genes, mostly the β-catenin and adenomatous polyposis coli genes [8,9]. Apart from FAP and Gardner's syndrome, some journals have also demonstrated an association between desmoid tumors and Crohn's disease [10]. In this case report, we see a rare occurrence of a desmoid tumor associated with an umbilical hernia in a male patient.

## Case presentation

A 52-year-old male presented to our outpatient department complaining of swelling in the umbilical region for the past three years, which was insidious in onset and gradually progressing in size to attain the current size; no sudden increase in size was seen. The swelling was initially completely reducible but partially reducible at the time of presentation. He had a history of intermittent dragging type of pain around the swelling for the past two weeks with no aggravating or relieving factor. There was no history of trauma, nausea, vomiting, difficulty passing stools, or flatus. There were no known comorbidities; he was a nonsmoker and a nonalcoholic. His diet and bladder habits were regular, with no loss of appetite or weight and no previous treatment or surgical history. There was no history of any malignancy in the family.

**Figure 1:**
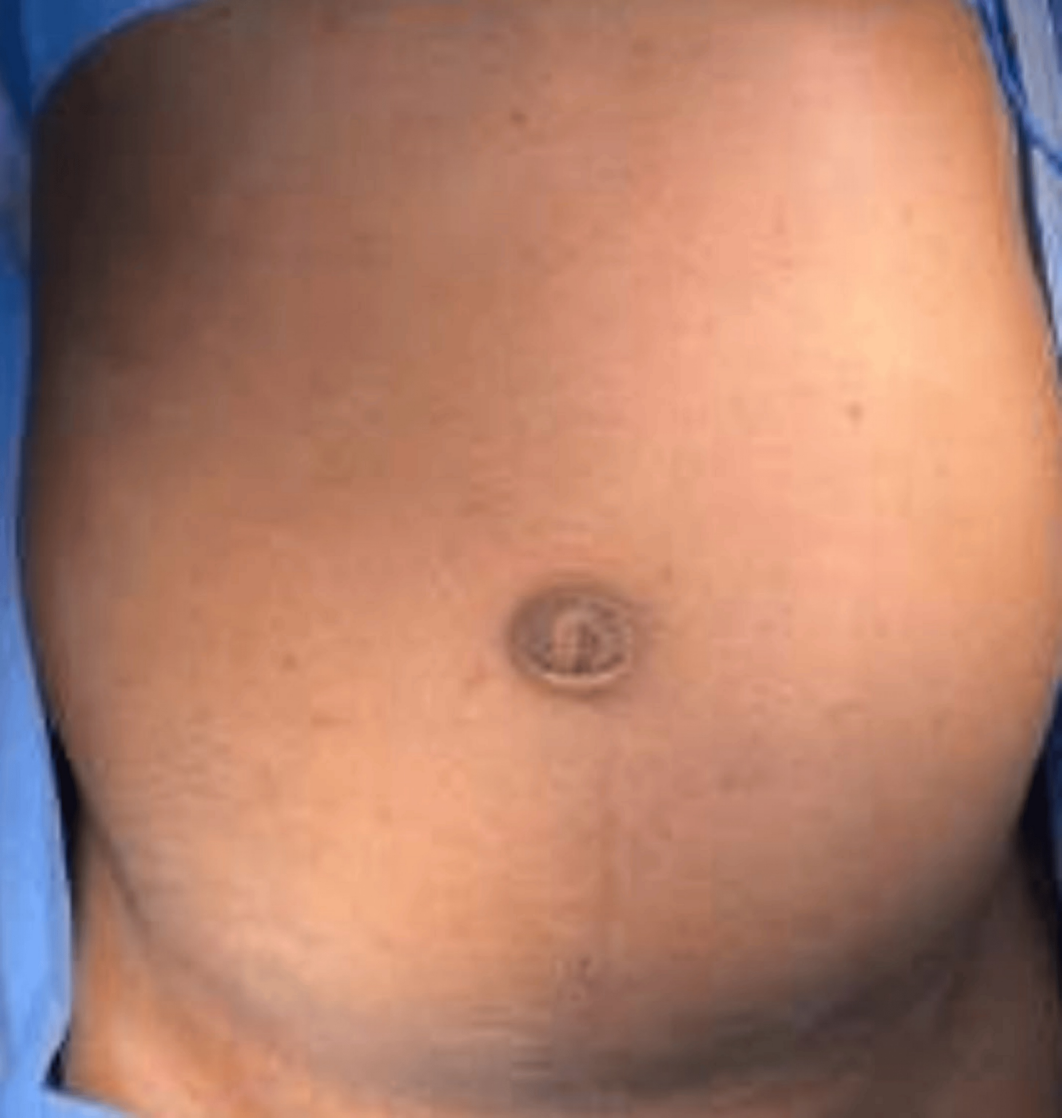
Gross picture of the abdomen of the patient showing a partially reduced umbilical hernia with a fullness seen in the infraumbilical region

On examining the patient, while standing up, there was a 4 x 3 cm umbilical swelling with noticeable cough impulse that reduced to a 2 x 1 cm swelling on lying down. On contracting the anterior abdominal wall, the umbilical defect was apparent. Further examination revealed another firm, immobile swelling of 10 x 8 cm in the infraumbilical region, extending from just below the umbilicus up to the pubic bone (Figure 1). On the head-raising test and leg-raising test, the swelling was almost completely reduced. The skin over both the swelling was normal with no abnormalities. No visible intestinal peristalsis or pulsation was seen either. Normal bowel sounds were heard. Other hernial orifices were normal, and external genitalia and per-rectal examination did not show any abnormality. Based on clinical examination, an initial provisional diagnosis was an umbilical hernia and an anterior abdominal wall mass, possibly hamartoma, lymphoma, gastrointestinal stromal tumor, neurofibroma, or desmoid tumor.

**Figure 2:**
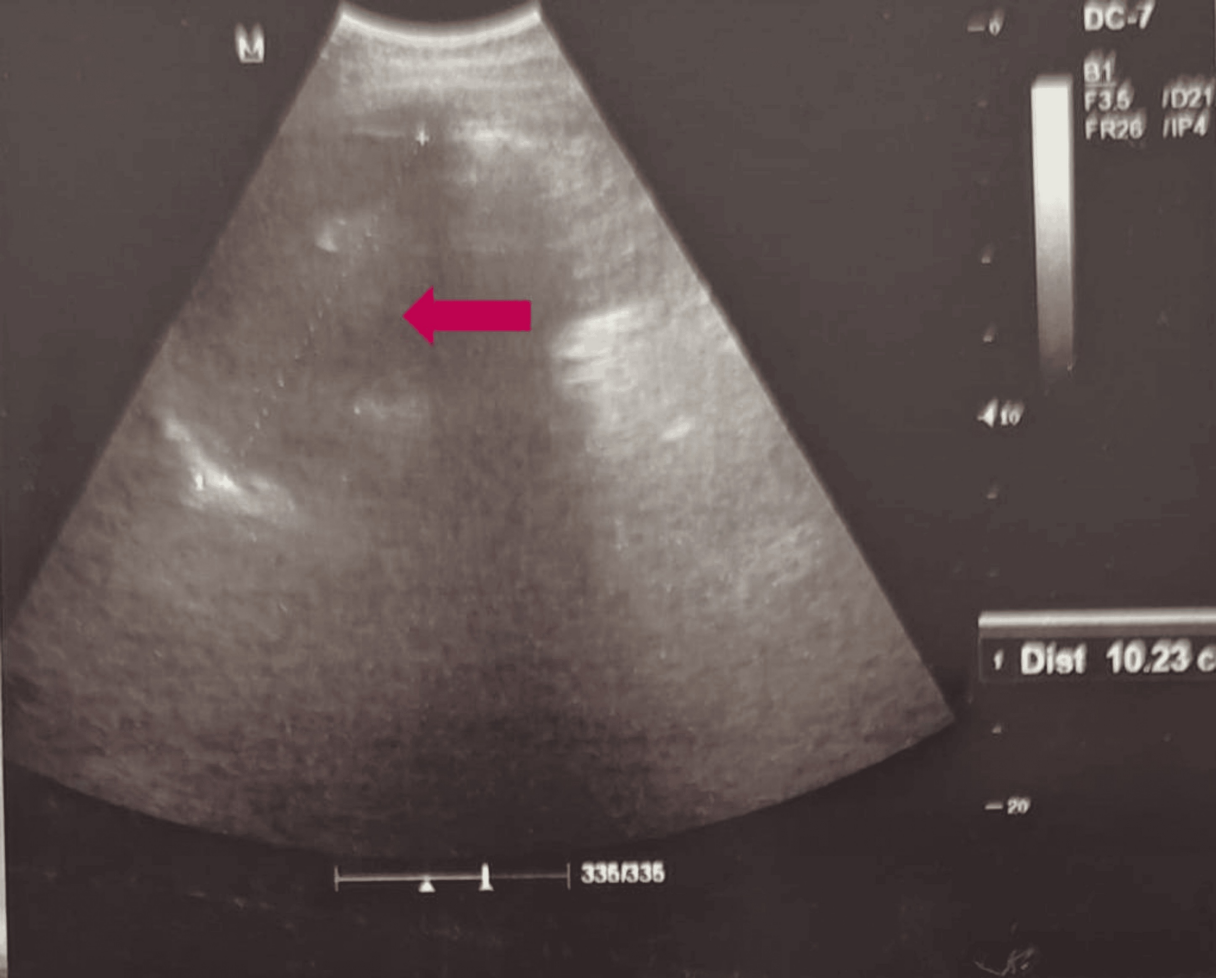
USG of the abdomen showing a heteroechoic lesion (red arrow) located posterior to the umbilicus USG: ultrasonogram

With initial evaluation, the ultrasonogram (USG) of the abdomen (Figure 2) showed a 1.5 cm defect in the umbilical region with an ill-defined infraumbilical heteroechoic lesion in the left paramedian region with mild internal and peripheral vascularity, abutting the urinary bladder posteroinferiorly, with grade II prostatomegaly.

**Figure 3:**
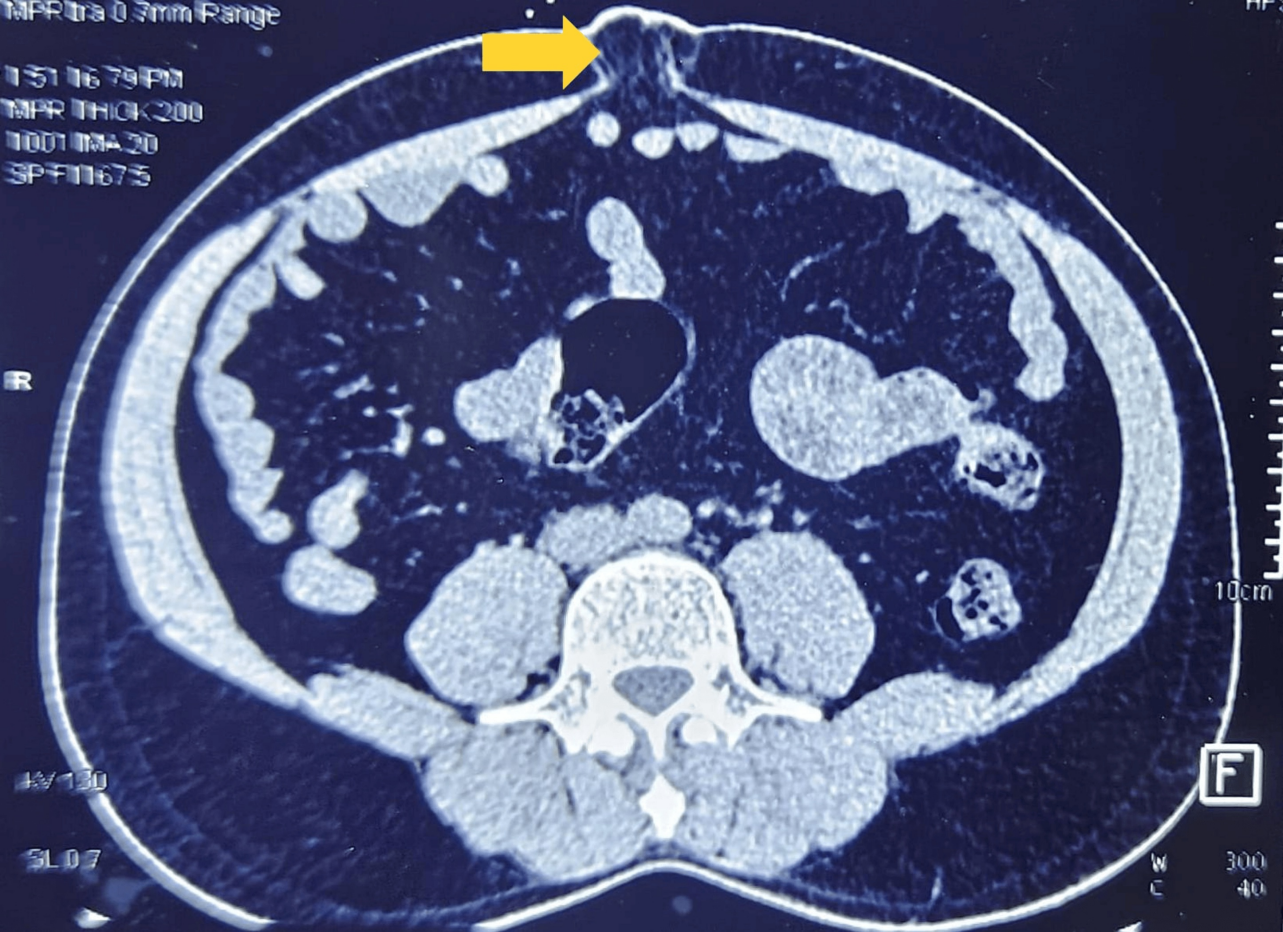
Transverse section of the abdominal CT scan, highlighting the presence of an umbilical hernia (yellow arrow) CT: computed tomography

**Figure 4:**
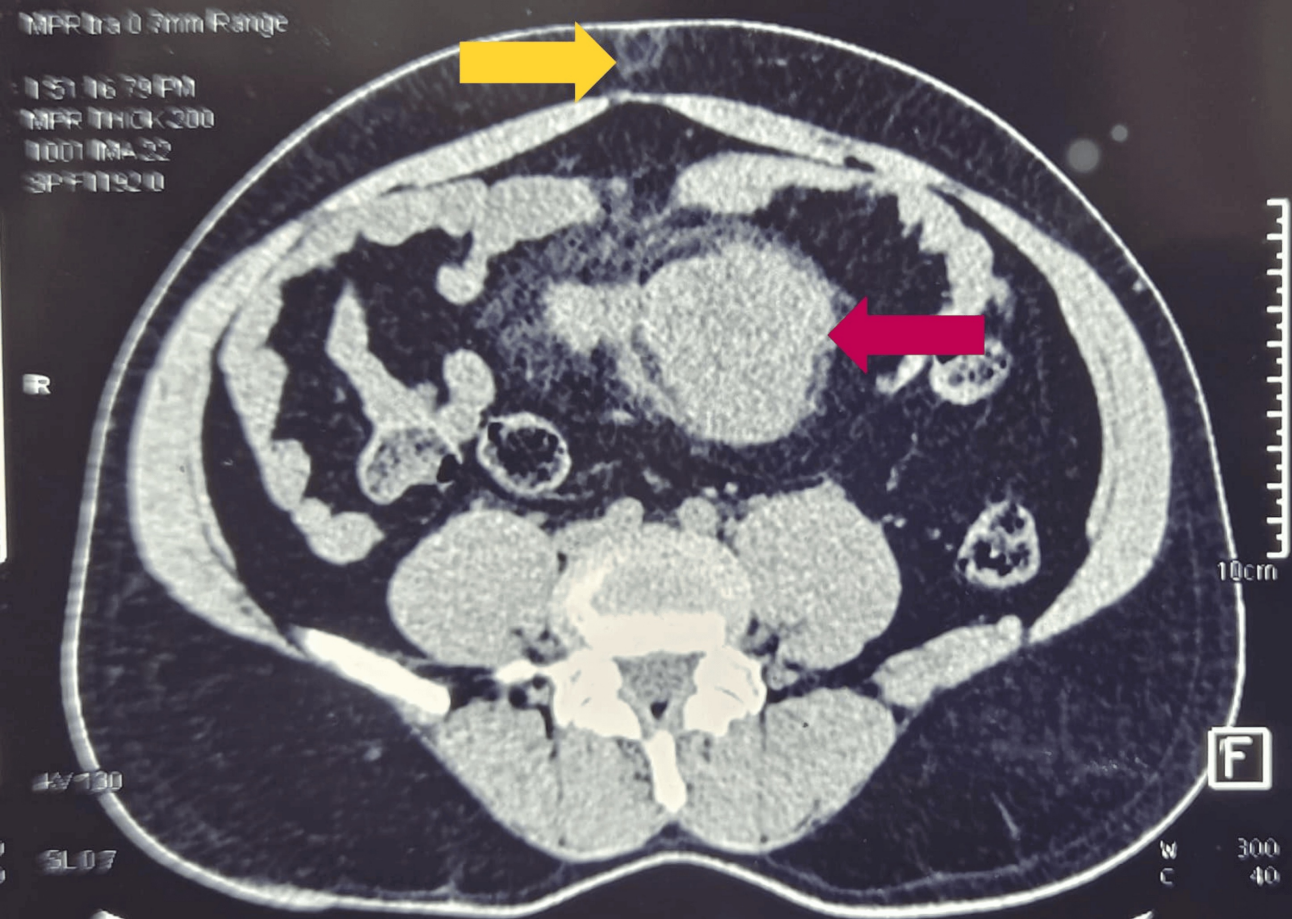
Transverse section of the abdominal CT scan, showing the umbilical hernia (yellow arrow) and the tumor (red arrow) CT: computed tomography

A follow-up computed tomography (CT) abdomen transverse section showed the umbilical hernia (Figure 3) along with a well-defined soft tissue lesion of size 13.5 x 8.8 x 13.2 cm in the lower anterior pelvis infra umbilically, a possible desmoid tumor or inflammatory pseudotumor (Figure 4), and prostatomegaly.

**Figure 5:**
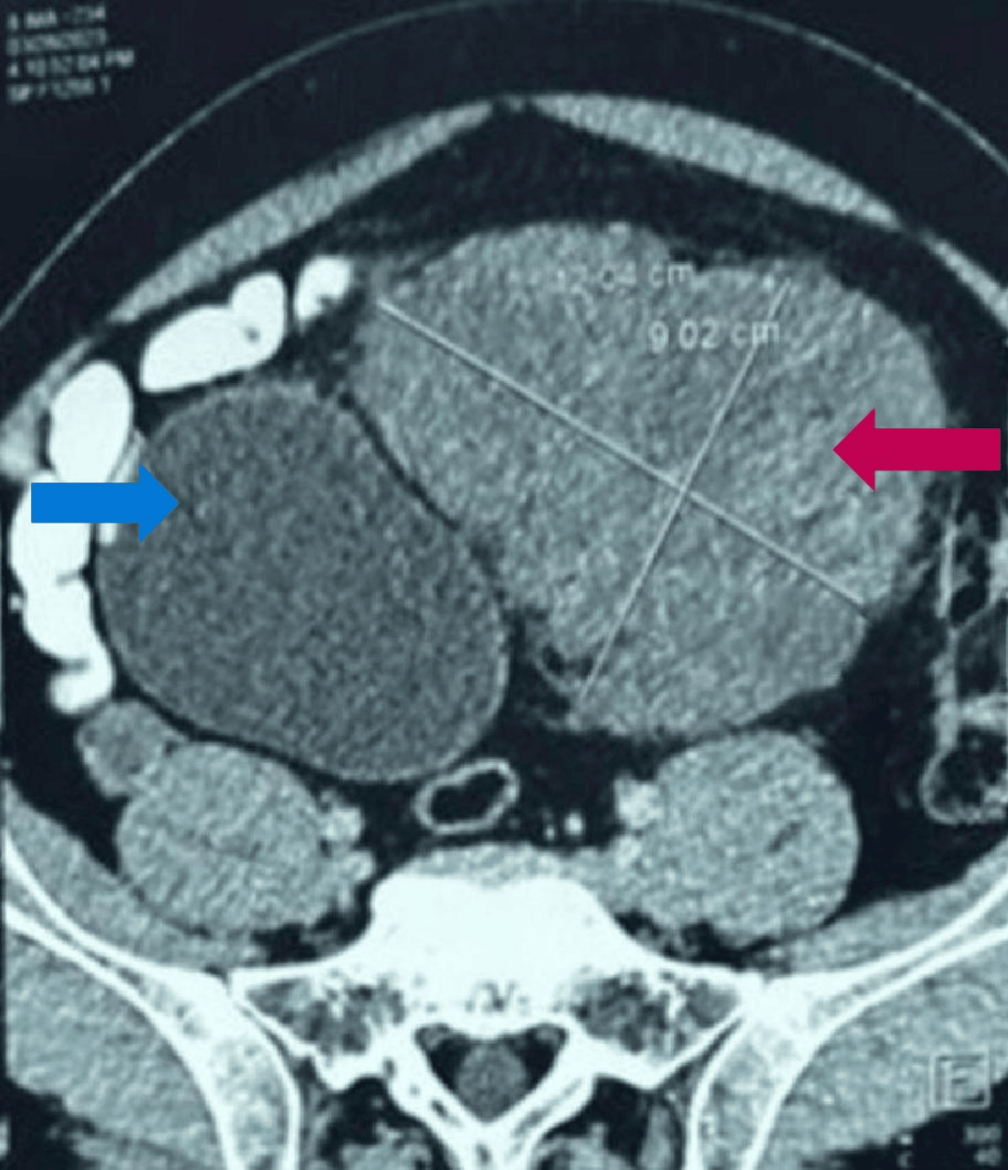
Transverse section of the abdominal CT scan, showing the tumor (red arrow) arising from posterior to the anterior abdominal wall without any infiltration to the urinary bladder (blue arrow) CT: computed tomography

**Figure 6:**
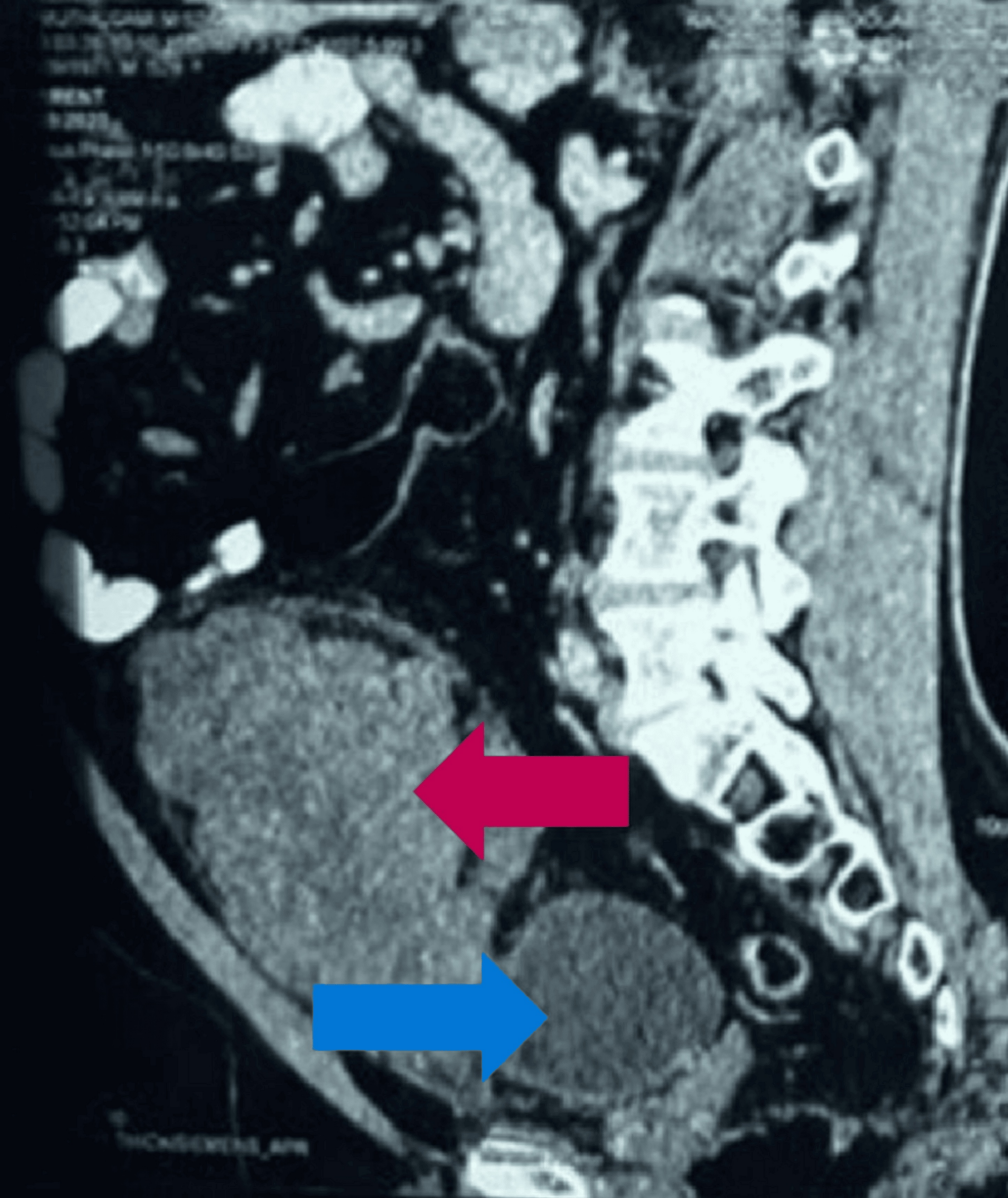
Sagittal section of the CT abdominal scan, showing the tumor (red arrow) arising from posterior to the anterior abdominal wall without any infiltration to the urinary bladder (blue arrow) CT: computed tomography

The CT abdomen transverse section (Figure 5) also showed the tumor abutting the urinary bladder, which was confirmed by the sagittal section (Figure 6).

**Figure 7:**
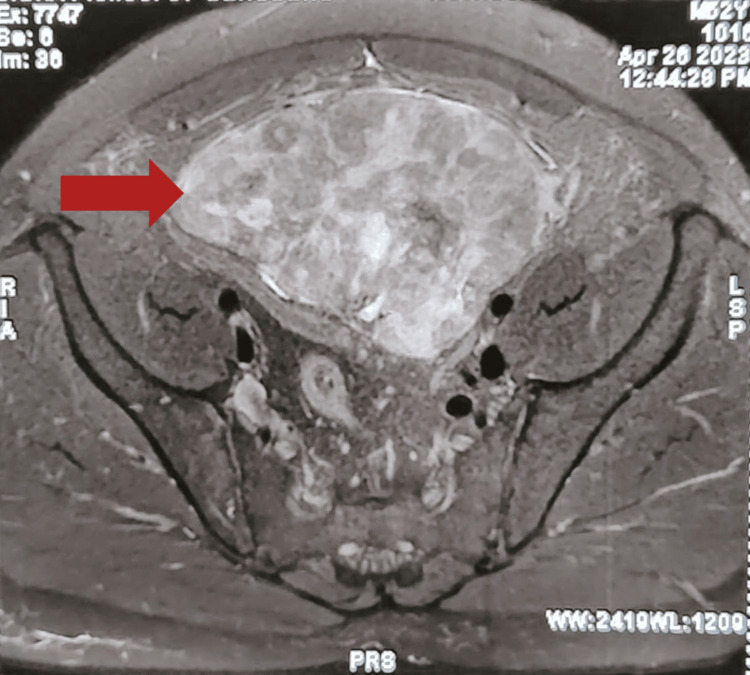
Transverse section of MRI abdominal scan, showing the tumor (red arrow) arising posterior to the anterior abdominal wall without infiltrating the urinary bladder MRI: magnetic resonance imaging

**Figure 8:**
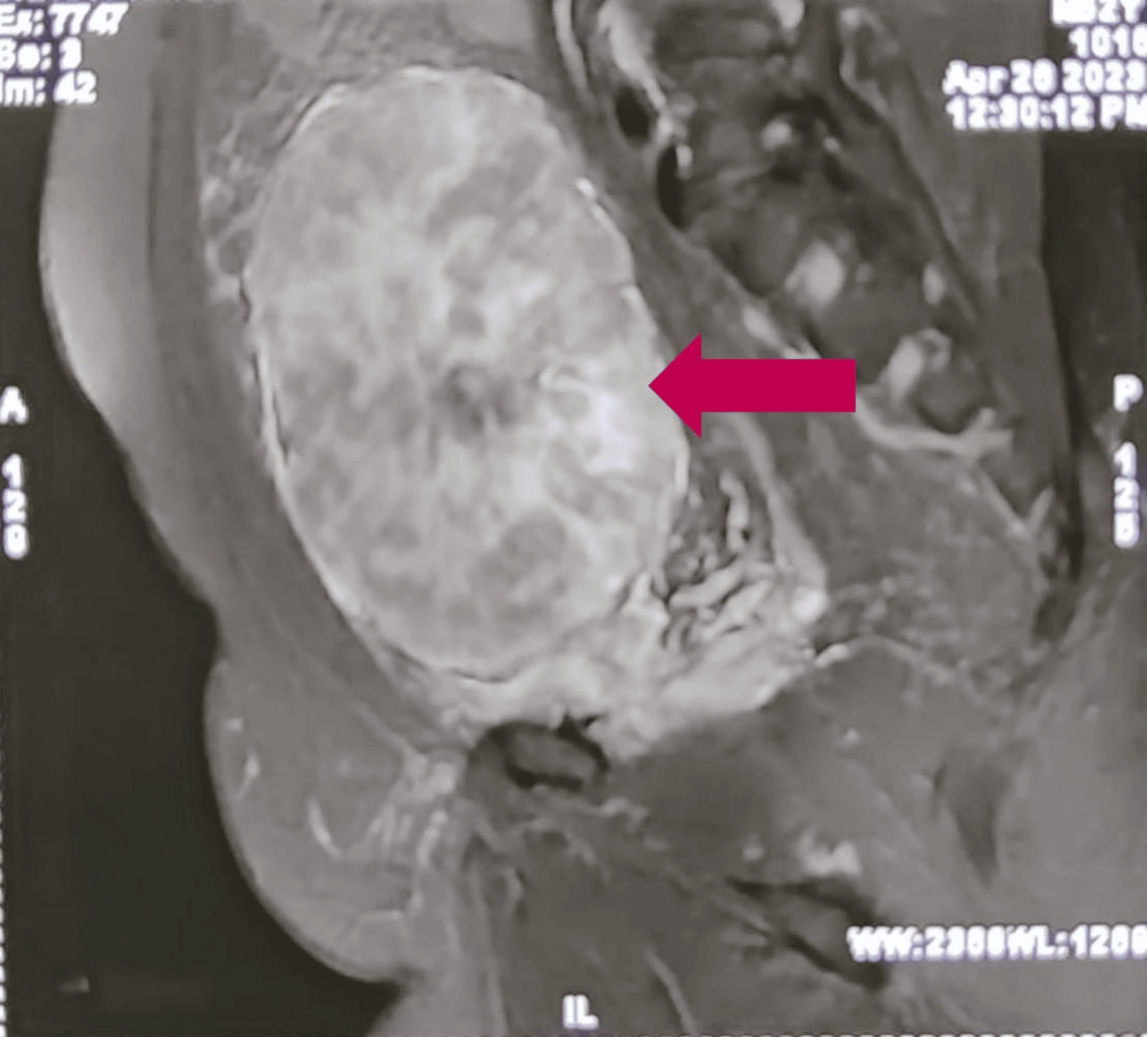
Sagittal section of the MRI abdominal scan showing the tumor (red arrow) arising possibly from the posterior rectus sheath, located in the preperitoneal space MRI: magnetic resonance imaging

To find any possible invasion of the tumor from the anterior abdominal wall, magnetic resonance imaging (MRI) of the abdomen was also done. The MRI of the abdomen in the transverse section (Figure 7) showed the tumor arising posterior to the anterior abdominal wall without infiltrating the urinary bladder, and the sagittal section (Figure 8) showed no extent of the tumor apart from the anterior abdominal wall but located in the preperitoneal space, possibly from arising from the posterior rectus sheath.

A USG-guided biopsy was done, which showed a benign spindle cell lesion and possible desmoid tumor. Colonoscopy was done to look for colonic polyps or ulcers to rule out FAP and Crohn's disease, respectively, which were negative. After discussing the case and the histopathological examination (HPE) report with the pathologist, we were advised to do a per-operative frozen section for tumor clearance. The patient elaborated on the rare occurrence of the type of tumor, and he consented to surgery.

**Figure 9:**
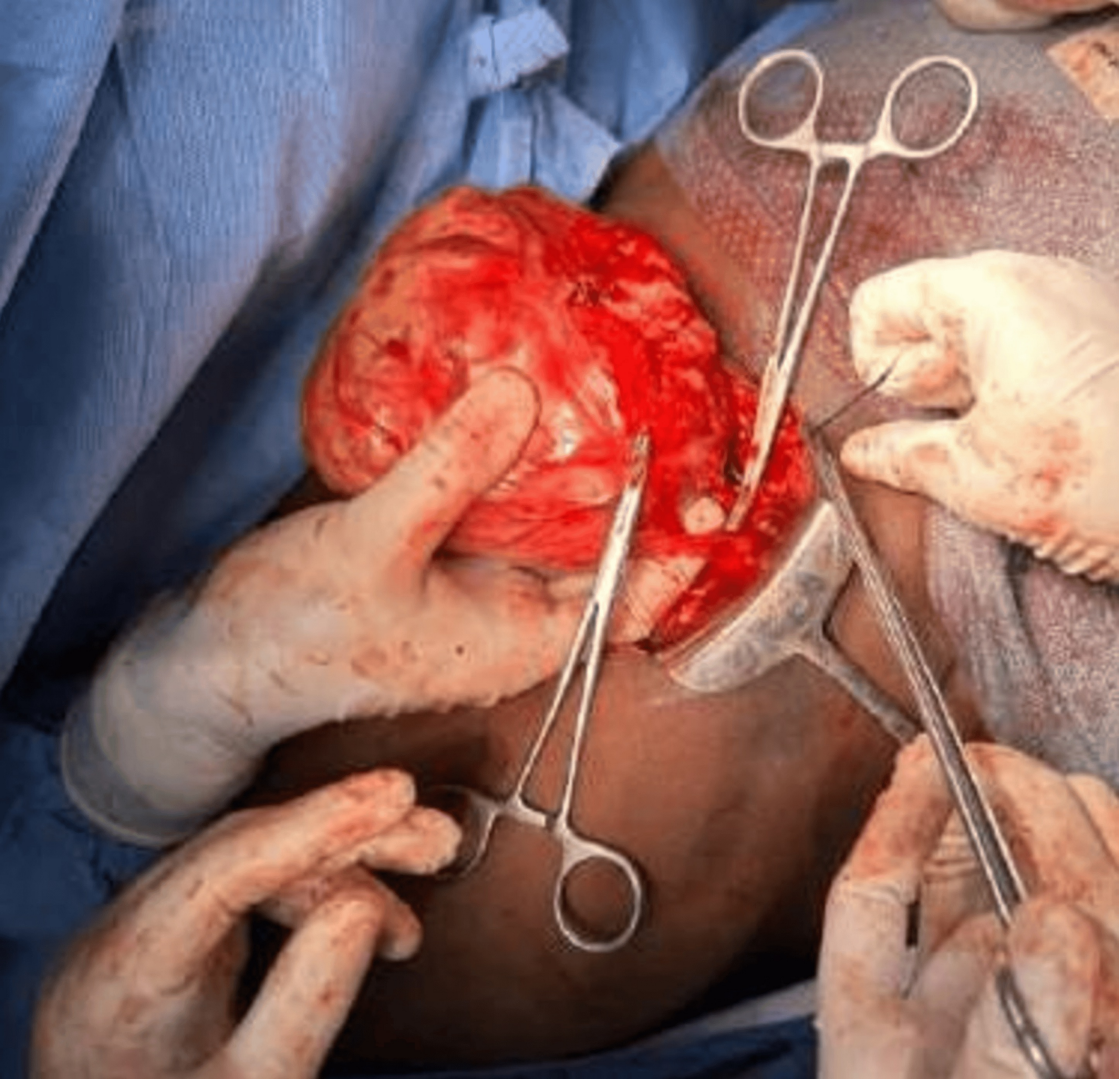
Per-operative picture showing the tumor being excised from the posterior rectus sheath with the feeder vessel being clamped below the tumor

Per-operatively, a lower midline laparotomy incision was made, the sac was first dissected from the umbilicus, and the rectus was partially opened. The sac's adhesion to the superior margin of the tumor was separated. The contents of the hernia were a small bowel and omentum. The tumor arising from the anterior abdominal wall muscles and posterior rectus sheath in the preperitoneal space extending up to the hypogastrium was found free of adhesions to the peritoneum or the urinary bladder wall. A feeder vessel noted posterior to the mass was identified and ligated (Figure 9). The tumor was excised completely with about 1 cm margin along with the posterior rectus sheath, marked, and sent for the frozen section for clearance. All margins were negative for malignant cells. Mesh placement was deferred, and umbilical herniorrhaphy was done since desmoid tumors have a high recurrence rate. The postoperative period was uneventful.

**Figure 10:**
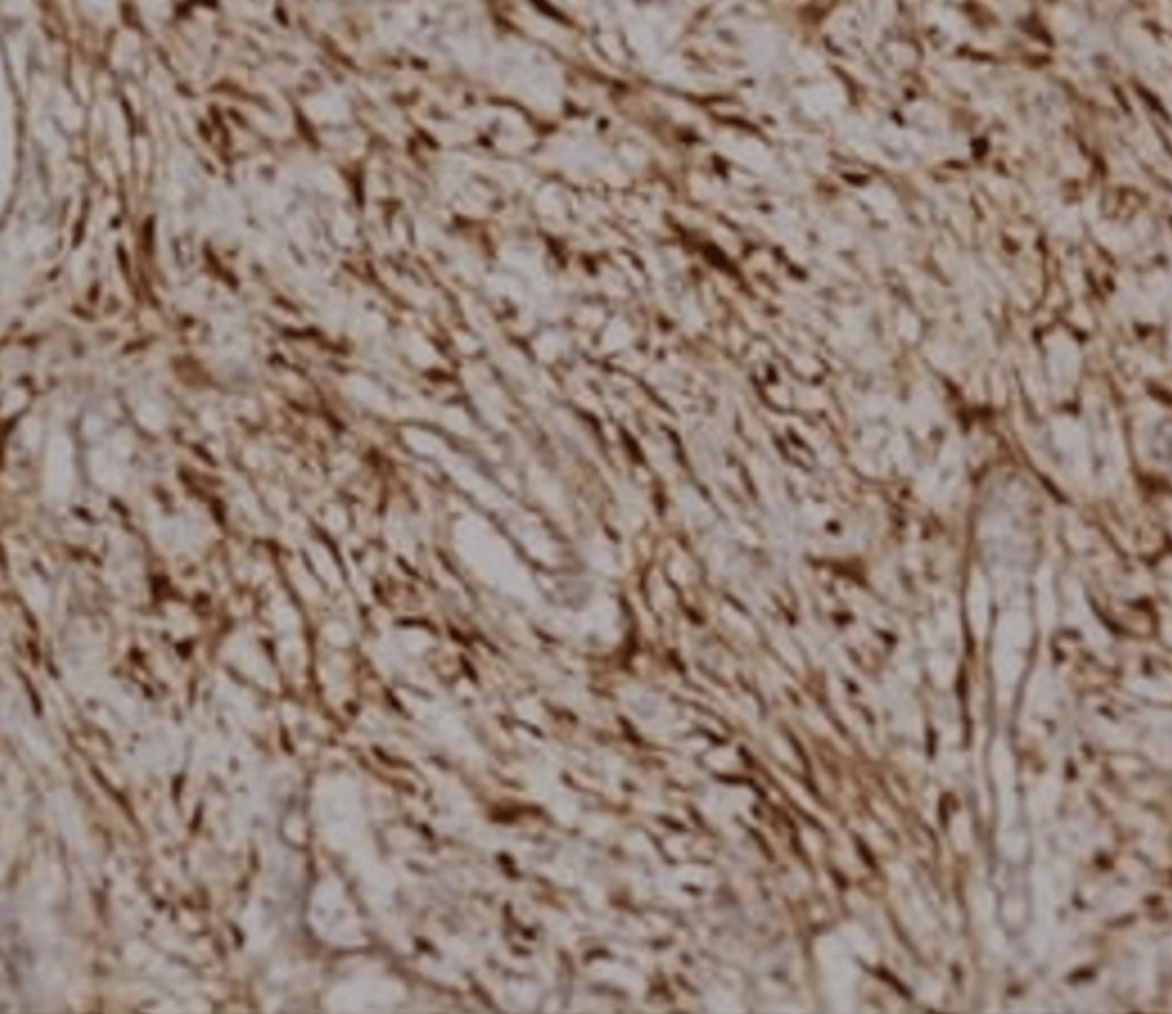
Immunohistochemistry, revealing nuclear accumulation in beta catenin in the slide

Postoperatively, the HPE report came out as desmoid fibromatosis. On immunostaining, nuclear accumulation of β-catenin was seen (Figure 10). APC mutation was weakly positive.

The medical oncologist discussed the case and advised chemotherapy. Postoperatively, the patient was discharged with no complications. The patient is on follow-up and has yet to be started on chemotherapy.

## Discussion

Desmoid tumors, also known as aggressive fibromatosis, are benign but locally aggressive tumors arising from the myoaponeurotic layer of the abdomen commonly seen in the anterior abdominal wall, particularly in the infraumbilical region in the hypogastrium. It is not very common to see desmoid tumors in males since the male-to-female ratio is about one to four. These tumors often undergo myxomatous changes but never sarcomatous changes, unlike fibromas. The first line of treatment that is widely done is surgical excision of the tumor along with postoperative chemotherapy. However, certain studies have also shown that neoadjuvant chemotherapy using doxorubicin and radiotherapy has also shown to be options alongside surgery only or surgery with chemotherapy [11]. In tumors progressing aggressively, other treatments recommended [12] are antiestrogens, nonsteroidal anti-inflammatory drugs, tyrosine kinase inhibitors, and low-dose chemotherapeutic agents such as methotrexate, vinblastine, and vinorelbine [13].

This case report emphasizes a few of the many challenges faced by a clinician on a patient's self-awareness, clinical examination, possible differential diagnosis, treatment choices for complicated cases, and uncertainty about the disease course. Due to the rarity of such presentation, no approved and established treatment options are available, though varied treatment choices are recommended based on clinical studies. For most patients, surgery is never the first option since active surveillance can be done. However, for complicated cases like these, a multidisciplinary team with experienced doctors, including surgeons, radiologists, pathologists, and oncologists, is needed to ensure the appropriate normal functioning of the patient [14]. Although some studies have shown that the chances of recurrence may vary based on the patients' age, size, and location of the tumor [15], there has been no established treatment regimen to decrease the high recurrence rate. But the end-goal of all these to be achieved is to improve the quality of life for the patient in the long term [13].

## Conclusions

Desmoid tumors are rare, locally aggressive benign tumors that arise in the body. The choice of treating this tumor ranges from active surveillance to tumor excision with postoperative chemotherapy. In our case, when a hernia with a semiemergency stage is present with the tumor, a combined herniorrhaphy with tumor excision with clearance and following postoperative chemotherapy will be needed. Herniorrhaphy was considered since if the tumor recurs, the hernia mesh would not intervene or inhibit the surgeon in the following surgery if needed.
